# Insulin-Related Liver Pathways and the Therapeutic Effects of Aerobic Training, Green Coffee, and Chlorogenic Acid Supplementation in Prediabetic Mice

**DOI:** 10.1155/2022/5318245

**Published:** 2022-02-24

**Authors:** Milad Abdollahi, Sayyed Mohammad Marandi, Kamran Ghaedi, Zahra Safaeinejad, Fatemeh Kazeminasab, Samaneh Shirkhani, Mohammad Hossein Sanei, Parsa Rezvanian, Mohammad Hossein Nasr-Esfahani

**Affiliations:** ^1^Department of Exercise Physiology, Faculty of Sport Sciences, University of Isfahan, Iran; ^2^Department of Cell and Molecular Biology and Microbiology, Faculty of Biological science and Technology University of Isfahan, Iran; ^3^Department of Animal Biotechnology, Cell Science Research Center, Royan Institute for Biotechnology, ACECR, Iran; ^4^Department of Physical Education and Sport Sciences, Faculty of Human Sciences, University of Kashan, Kashan, Iran; ^5^Acquired Immunodeficiency Research Center, Isfahan University of Medical Sciences, Isfahan, Iran

## Abstract

**Background:**

The liver controls blood glucose levels via regulation of anabolic (glycogen synthesis and gluconeogenesis) and catabolic (glycolysis and glycogenolysis) processes through activation of the PI3K-AKT signalling pathway. The aim of this study was to assess the effect of aerobic training, green coffee, and chlorogenic acid supplementation on glucose metabolism-regulating pathways in prediabetic mice.

**Methods:**

C57BL/6 mice were exposed to a high-fat diet and physical activity limitation to induce a state of prediabetes. After 12 weeks, mice were fed a high-fat diet compared to the control mice. The prediabetic mice were further treated with either green coffee, chlorogenic acid, or training or combinations of the same for 10 weeks. At the end of the experimental period, metabolic data (FBG, GTT, HOMA for IR, plasma level of insulinfrom systematic, AST, and ALT assessed into blood), histopathologic, and analysis of gene and protein expressions were obtained for target tissues.

**Results:**

Training along with green coffee and chlorogenic acid supplementation improved complications of prediabetes including weight gain and elevated fasting blood glucose and plasma insulin levels. These effects were associated with the changes in mRNA levels of genes important in hepatic glycogen synthesis (GYS2), glucogenesis (PCK and G6PC2), and glycolysis (GK, PK, and PFKL).

**Conclusion:**

The training in conjunction with green coffee or chlorogenic acid is effective in the prevention of prediabetes in mice. As these interventions are relatively inexpensive and safe application to individuals with prediabetes appears warranted.

## 1. Introduction

Prediabetes (PD) is a metabolic disorder characterized by higher than normal and less than diabetic levels of blood glucose [1, 2]. Impaired fasting glucose (IFG) and glucose tolerance (IGT) tests assist in making the diagnosis [3, 4]. In prediabetic patients, normal levels of fasting blood glucose and glucose tolerance test are 100-125 mg/dl and 145-199 mg/dl, respectively. Prediabetics have 3-10 times the risk of progressing to type 2 diabetes mellitus (T2DM) compared to nonprediabetics [5]. The primary cause of PD is insulin resistance in the muscle, liver, and adipose tissues. This progressive resistance to insulin leads to degradation in pancreatic beta-cell function and decreased insulin secretion and T2DM. Obesity is considered a primary factor accounting for insulin resistance. This is important, as current lifestyle factors such as sedentary routines and consumption of high-calorie diets promote weight gain and obesity [6, 7].

**Figure 1:**
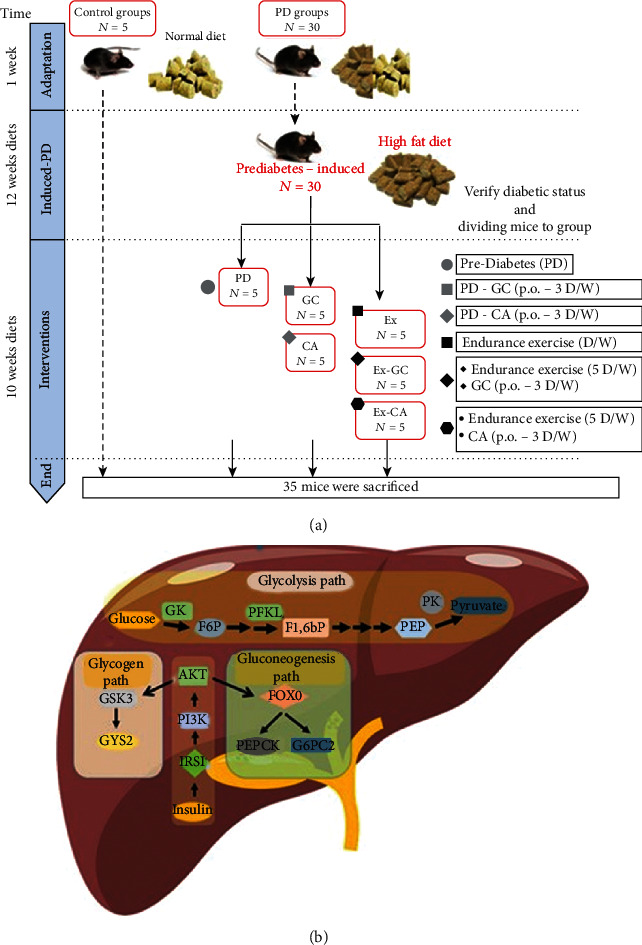
Research design. (a) Methodology. Induction of prediabetes has been shown during interventions (first intervention [12 weeks, mice were divided into control (Ctrl) and prediabetic (PD) groups]) and treatment (second intervention [10 weeks, PD were randomly divided into 6 groups: prediabetes group (PD), 2 prediabetes groups treated with green coffee (PD/GC), 3 prediabetes group treated with chlorogenic acid (PD/CGA), 4 prediabetes groups treated with aerobic training (PD/EX), 5 prediabetes group treated with aerobic training and green coffee (PD/EX.GC), and 6 prediabetes group treated with aerobic training and chlorogenic acid (PD/EX.CGA).]). All mice were euthanized at the end of the 22^nd^ week. (b)Measurement. Insulin-related and glycolysis pathways.IRS1/2, insulin receptor substrate 1; PI3K, phosphoinositide-3 kinase; AKT, protein kinase B; GSK3, glycogen synthase kinase 3; GYS2, glycogen synthase 2; FOXO1, forkhead box protein O1; G6PC2, glucose-6-phosphatase 2; PEPCK, phosphoenolpyruvate carboxykinase. GK, glucokinase; F6P, fructose 6-phosphate; PFKL, phosphofructokinase, liver type; F16BP, fructose 1,6-bisphosphate; PK, pyruvatekinase.

Insulin is secreted from beta cells of the pancreas into the portal vein. Thus, unlike other organs that receive insulin from systemic blood circulation, the liver is exposed to 2-3 times more insulin to suppress glycogenolysis and glucogenesis. The importance of insulin on whole-body glucose homeostasis in the liver is well documented [8, 9]. In hepatocytes, insulin acts through the PI3K/AKT signalling pathway. In hepatocytes, insulin binds to its receptors resulting in IRS1/2 phosphorylation activating the downstream target PI3K/AKT. Glycogen synthase kinase-3 (GSK3), transcription factor forkhead box O1 (FOXO1), and glucokinase (GK) are AKT pathway substrates [10, 11]. On the one hand, insulin resistance and obesity, hepatic IRS1/2 undergoes serine phosphorylation to limit AKT activation and disrupt glycogen synthesis, thus promoting glycogenolysis and gluconeogenesis [12]. On the other hand, glycolysis is also disrupted by insulin resistance [12]. Glycogen synthase 2 (GYS2), as the main enzyme in glycogen synthesis, is regulated by insulin through control of transcription and cytoplasmic to nucleus transit [9](Figure 1).

Lifestyle routines including diet, training, and maintenance of an ideal body weight provide protection from PD [1, 13, 14]. Coffee is widely consumed and has potential therapeutic properties. Green coffee (GC) is unroasted, increasing in use, is thought to have antioxidant and anti-inflammatory properties, and has been propped as a treatment for certain diseases. Green coffee is rich in phytochemicals such as chlorogenic acid (CA) that is believed to be the main source of its beneficial properties [15–18].

The American Diabetes Association recommends that adults with diabetes perform moderate to vigorous aerobic training to reduce the prevalence of diabetes-related complications such as cardiovascular and renal disease [19, 20]. As such, training remains a cornerstone, albeit not adhered to, in the prevention and treatment of T2DM. There is an abundance of studies on the effect of green coffee and its active ingredient chlorogenic acid on diabetes. However, no report has assessed these natural products in combination with training on hepatic glycogen storage in PD. In the current study, we evaluated the simultaneous effects of aerobic trainingwith green coffee and chlorogenic acid on glycogen synthesis, glycolysis, and gluconeogenesis in the liver of PD mice.

## 2. Material and Methods

### 2.1. In Vivo Experiments

This study was approved by the Ethics Committee of Royan Institute (ethics code: IR.ACECR.ROYAN.REC.1399.075). All animals were treated according to the Animal Ethics Committee of Royan Institute's recommendations.

Four-week-old male C57BL6 mice, weighing 12–14 g, were used in this study. Mice were housed in pathogen-free barrier facilities under controlled temperature (23°C ± 3°C) and humidity with a 12-h light/dark cycle. Access to food and water was ad libitum. After 1 week of adaptation, the mice were randomly divided into two groups: control (Ctrl; *n* = 5) and high-fat diet (HFD; *n* = 30) mice. Mice in the Ctrl group received a standard diet (containing carbohydrates 47.7%, fat 12.5%, and protein 20.5%) while mice in the HFD group received a diet containing 25% carbohydrates, 60% fat, and 15% protein for 12 weeks. Particularly, the fat ingredients in HF diets are saturated. After ensuring the emergence of PD, the HFD group was divided into six subgroups (*n* = 5): prediabetic mice (PD group), prediabetic mice treated with green coffee (200 mg/kg, PD/GC group), prediabetic mice treated with chlorogenic acid (100 mg/kg, PD/CGA), prediabetic mice which underwent training (PD/EX), prediabetic mice treated with green coffee+training (PD/EX. GC), and prediabetic mice treated with chlorogenic acid+training (PD/EX. CGA) [21–24].

**Figure 2:**
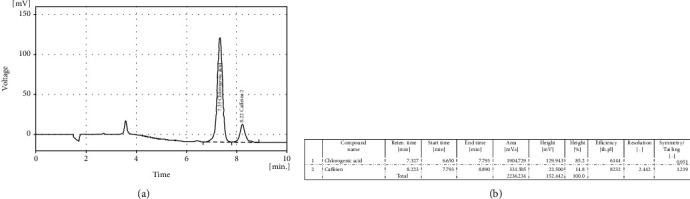
HPLC chromatogram of CGAs in green coffee bean extract (BSK). (a) the percentages of chlorogenic acid and caffeine in green coffee. (b) the biochemical characteristics of chlorogenic acid and caffeine in green coffee.

Green coffee tablets were purchased from BSK (Zist Takhmir, Tehran-Iran). These tablets are a natural product made from 400 mg of standardized green coffee bean extract powder containing 2% caffeine and 50% chlorogenic acid, which these main substates analyzied by HPLC. Briefly, HPLC analysis of CGAs in green coffee bean extract (BSK) was performed using the HPLC-diode array detector gradient system (Agilent 1090 series) (Figure 2). Green coffee and CGA were administered as a gavage supplement, three times per week for 10 weeks.

During the experiment period, the mice were weighed every 7 days. At the end of the experimental period, animals were euthanized after a 12-h overnight fast under xylazine and ketamine anaesthesia. Serum and liver tissues were immediately stored at −80°C for further analysis. Figure 1 demonstrates the flowchart of the study.

### 2.2. Biochemical Analyses

Fasting blood glucose (FBG) and glucose tolerance tests (GTT) were performed at the end of the 12th and 22^nd^ week of the intervention using a glucometer (Alpha TRAK glucometer, Zoetis, US) from a tail prick. For GTT, mice were fasted for 6 hours and then gavaged with 200 *μ*l glucose solution. Blood glucose was measured at 0, 30, 60, 90, and 120 min later. The plasma level of insulin was determined with an Ultra-Sensitive Mouse Insulin ELISA Kit (ALPCO 80-INSMS-E01, Keewaydin Drive, USA) according to the manufacturer's instructions. Plasma aspartate aminotransferase (AST) and alanine aminotransferase (ALT) levels were measured using Mouse AST and ALT ELISA Kits (ELISA Kit- CSB-E12649m, UK and ELISA Kit- MBS016898, respectively, Abcam, USA, San Diego) according to the manufacturer's protocol.

### 2.3. Training Intervention

Aerobic training was carried out for 10 weeks (5 days/week). Briefly, all mice were acclimated to running on a treadmill (10 min, using different speeds) for 1 week. On day 1, an electric shock is applied to make the mice start running, after which they will run spontaneously. In other words, this method provides the sole external motivator through grid and mice quickly acquire that make maintain some distance from the grid when running [25]. Aerobic training was performed on a treadmill at low to moderate intensity (50%-60% MAV), 45 minutes per day for 5 days per week for 10 weeks, the exact time for training was between 10 p.m. and 11 p.m. The experiment was carried out in a quiet, well-ventilated room with low humidity at a temperature of 18 ± 2°C. The sedentary mice in the control group were treated similar to the training mice except they were not engaged in regular running. Each training session included 3 minutes of warm-up, 40 minutes of training, and 2 minutes of recovery. The initial training intensity was 15 m/min. The intensity was increased 2 m/minute every 2 weeks until it reached 23 m/min on the final week. Thus, the first and second week used an intensity of 15 m/min, the third and fourth week an intensity of 17 m/min, the fifth and sixth week an intensity of 19 m/min, the seventh and eighth week an intensity of 21 m/min, and the ninth and tenth week an intensity 23 m/min [26].

### 2.4. RT-qPCR Analysis

The total RNA isolation from the liver tissue was performed using the TRIzol reagent (Thermo Fisher Scientific, Waltham, MA, USA). In order to remove contaminating genomic DNA, samples were treated with DNase I (TaKaRa, Japan). mRNA was reverse transcribed with 1 *μ*g of total RNA using the cDNA synthesis kit (Biotechrabbit, Germany, Berlin) according to the manufacturer's instruction. RT-qPCR was performed with SYBR Green (TaKaRa, Japan) using an Applied Biosystems real-time PCR thermal cycler (Thermo Fisher Scientific, Waltham, MA, USA). The evaluation of gene expression was carried out according to the 2^−*ΔΔ*ct^ method. Accordingly, the relative gene expression was calculated according to 18 s rRNA as an internal control. Primer pairs were designed by the Beacon designer (Version 7.2, USA) and purchased from Micro-gene (Korea). The primer sequences are shown in Table 1.

### 2.5. Western Blot Analysis

Proteins were extracted from the tissue samples using the TRI reagent, according to the manufacturer's protocol. Equal amounts of each protein sample (30 *μ*g) were subjected to SDS PAGE and transferred to PVDF membranes. Membranes were blocked with a blocking buffer containing 10% skim milk (Millipore, USA) and 5% TBST. The membranes were probed with primary antibodies including anti-Akt antibody (1: 2000, ab30471, Elabscience, USA), anti-p-Akt (Thr 308) antibody (1 : 1000, sc271966, Santa Cruz, USA), anti-*β* actin antibody (1 : 1000, sc47778, Santa Cruz, USA), antiglycogen synthase 2 antibody (1 : 1000, sc390391, Santa Cruz, USA), and anti-phospho-glycogen synthase (Ser641) antibody (1 : 1000, Cell Signaling, USA) for 1.5 hours at room temperature. Subsequently, the membranes were incubated for 1 hour at room temperature with an appropriate secondary antibody: horseradish peroxidase- (HRP-) conjugated goat antimouse IgG (1 : 5000, Dako, P0447) or HRP-conjugated goat antirabbit IgG (1 : 16000, Santa Cruz, SC2301). Bands were visualized by an Amersham ECL Advance Western Blotting Detection Kit (GE healthcare). The activity of AKT protein was measured by comparing the level of phosphorylated AKT with the level of nonphosphorylated AKT. The ImageJ software (National Institutes of Health, Bethesda, MD, USA) was utilized for quantification of the intensity band.

### 2.6. Histological Studies

Immediately after the human euthanasia of the mice, the livers were removed, selected at random, sliced into small pieces, and were fixed in 10% buffered formalin and embedded in paraffin. Fixed tissues were then cut into 5-*μ*m thickness tissue sections. After deparaffinization and hydration, sections were stained with hematoxylin and eosin (H&E). The periodic acid-Shiff (PAS) was used to stain glycogen in the liver sections. Slides were observed using a light microscope.

### 2.7. Statistical Analysis

The statistical analyses were carried out using GraphPad Prism 8.4.3 software (GraphPad Software, San Diego, CA, USA).The paired sample *t*-test was performed to evaluate the prediabetic group compared to the control group. One-way analysis of variance (ANOVA) and Tukey's post-hoc test were used to determine statistical significance between all treatment groups. *p* − value < 0.05 depicts significant difference between samples. All experimental results are presented as mean ± SD.

## 3. Results

### 3.1. HFD Promotes Prediabetes in Mice

**Figure 3:**
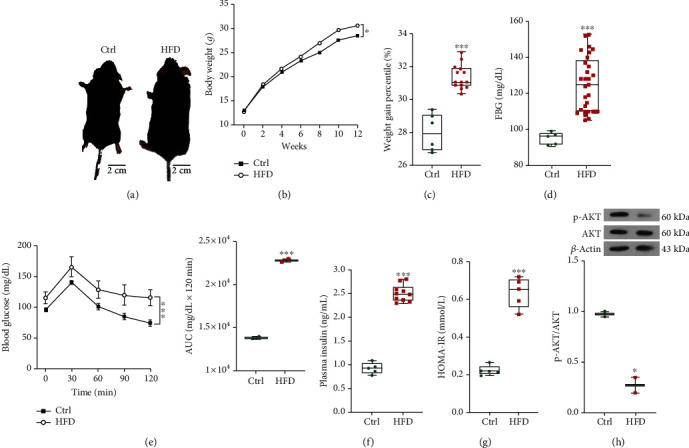
HFP promotes PD in mice. (a) Ctrl and HFD mice, (b and c) the percentage changes of bodyweight (12 weeks), and weight gain ([the final body weight (23 weeks) - the primary bodyweight (12 weeks)]). (d-g) Plasma Biochemical Analysis including (d and e) fasting blood glucose (FBG) concentration, glucose tolerance test (GTT), and the area under the curve (AUC) in Ctrl and HFD groups. (f) Plasma insulin concentration and (g) HOMA index in Ctrl and HFD groups. (h) Immunoblot analyses of liver lysates for protein level evaluations such as p-AKT and total AKT in Ctrl and HFD groups. All values are presented as mean ± SD. ^∗^ = *p* < 0.05 and ^∗∗∗^ = *p* < 0.001 indicate significant statistical differences in HFD compared to Ctrl mice.

**Figure 4:**
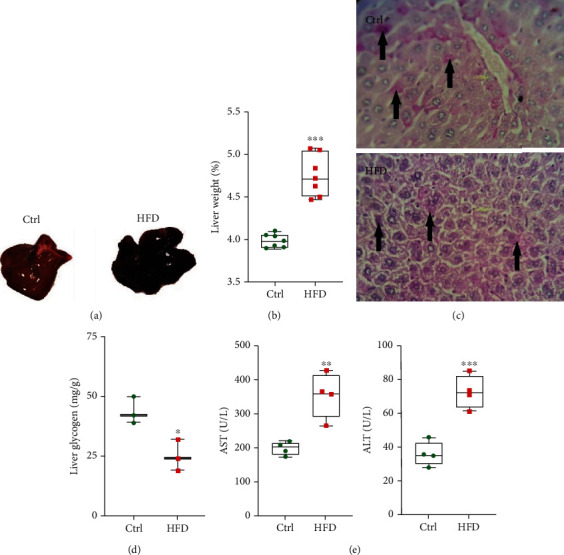
HFD promotes liver damage in mice. (a) Liver morphology of Ctrl and HFD mice. (b) The percentage change of liver weight (The ratio of liver to total body weight for Ctrl and HFD mice). (c) Histopathology of liver sections (PAS staining) from Ctrl and HFD mice (arrows indicate glycogen accumulations). (d) Quantitative assessment of hepatic glycogen (mg/g). (e) Systematic plasma levels of specific liver enzymes (AST and ALT ) in Ctrl and HFD mice. All values are presented as mean ± SD. ^∗^ = *p* < 0.05, ^∗∗^ = *p* < 0.01, and ^∗∗∗^ = *p* < 0.001 indicate significant statistical differences in HFD compared to Ctrl mice.

The animals given the HFD showed a significant increase in body weight and the percentage of weight gain (Figures 3(b) and 3(c)). Additionally, the animals demonstrated increases in FBS, GTT (Figures 3(d) and 3(e)), and plasma insulin levels compared to the mice receiving the ND (Figure 3(f)). Consistent with these changes, the HOMA-IR index was significantly elevated in the HFD-fed mice (Figure 3(g)). The Western analysis of hepatic protein expression demonstrated less AKT protein phosphorylation in organs from the HFD versus the ND group (Figure 3(h)). Interestingly, the liver morphology and histopathology were comparable among groups and not grossly abnormal (data not shown). However, the ratio of liver weight to total bodyweight was greater for the HFD-fed mice (Figures 4(a) and 4(b)). As expected, PAS staining was less in liver samples from the HDF-fed mice (Figures 4(c) and 4(d)). Finally, the analysis of plasma levels of AST and ALT found that the enzymes were significantly higher in mice receiving the HFD compared to the ND-fed animals (Figure 4(e)).

### 3.2. HFD Is Associated with Changes in mRNA Levels of Genes Involved in Hepatic Glycogen Synthesis, Gluconeogenesis, and Glycolysis

**Figure 5:**
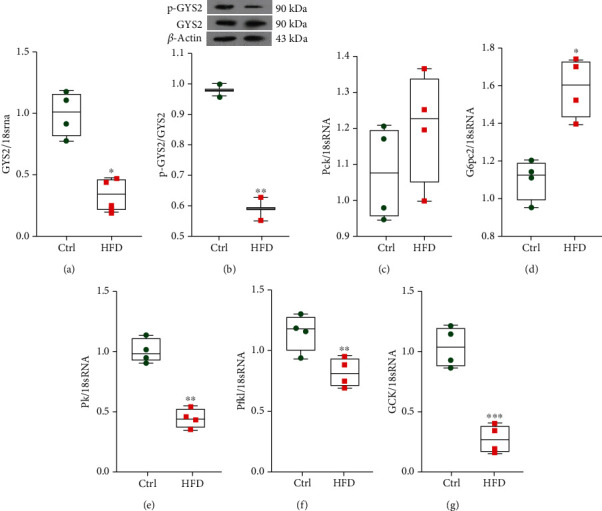
HFD is associated with changes in hepatic metabolic enzyme mRNA levels. (a) RT-qPCR analysis of Gys2 in the livers from the Ctrl and HFD mice. (b) Immunoblotting analyses of p-GYS2 and total GYS2 protein in the livers from the Ctrl and HFD groups, (c) RT-qPCR analysis of *G6pc2*, and (d) *Peck* mRNA in the livers from the Ctrl and HFD mice. (e–g) RT-qPCR analysis of *Pk*, Pfkl, and Gk mRNA in the livers from the Ctrl and HFD mice. All values are presented as mean ± SD. ^∗^ = *p* < 0.05, ^∗∗^ = *p* < 0.01, and ^∗∗∗^ = *p* < 0.001 indicate significant statistical differences in the HFD compared to Ctrl mice.

In order to investigate the possible causes of hyperglycemia and reduced liver glycogen content, hepatic mRNA levels of genes associated with glycogen synthesis, glycolysis, and gluconeogenesis were examined. The livers from HFD-fed mice showed reduced expression of Gys2 mRNA (Figure 5(a)). The Western analysis of hepatic protein expression demonstrated less GYS2 protein phosphorylation in organs from the HFD versus the ND group (Figure 5(b)). The mRNA levels of two gluconeogenesis enzymes, phosphoenolpyruvate carboxykinase (Pck1 = Peck and glucose-6-phosphatase catalytic subunit 2 (G*6pc2*), were increased in mice on the HFD compared to the mice on the ND, although in the case of Pck1was unchanged. (Figures 5(c) and 5(d)). Additionally, in the livers from the HFD mice, mRNA levels of glucokinase (*Gk*), pyruvate kinase (P*k*), and (P*fkl*) phosphofructokinase were decreased compared to the controls (Figures 5(e), 5(f), and 5(g)).

### 3.3. Training, Green Coffee, and Chlorogenic Acid Mitigated PD-Associated Changes in HFD-Fed Mice

**Figure 6:**
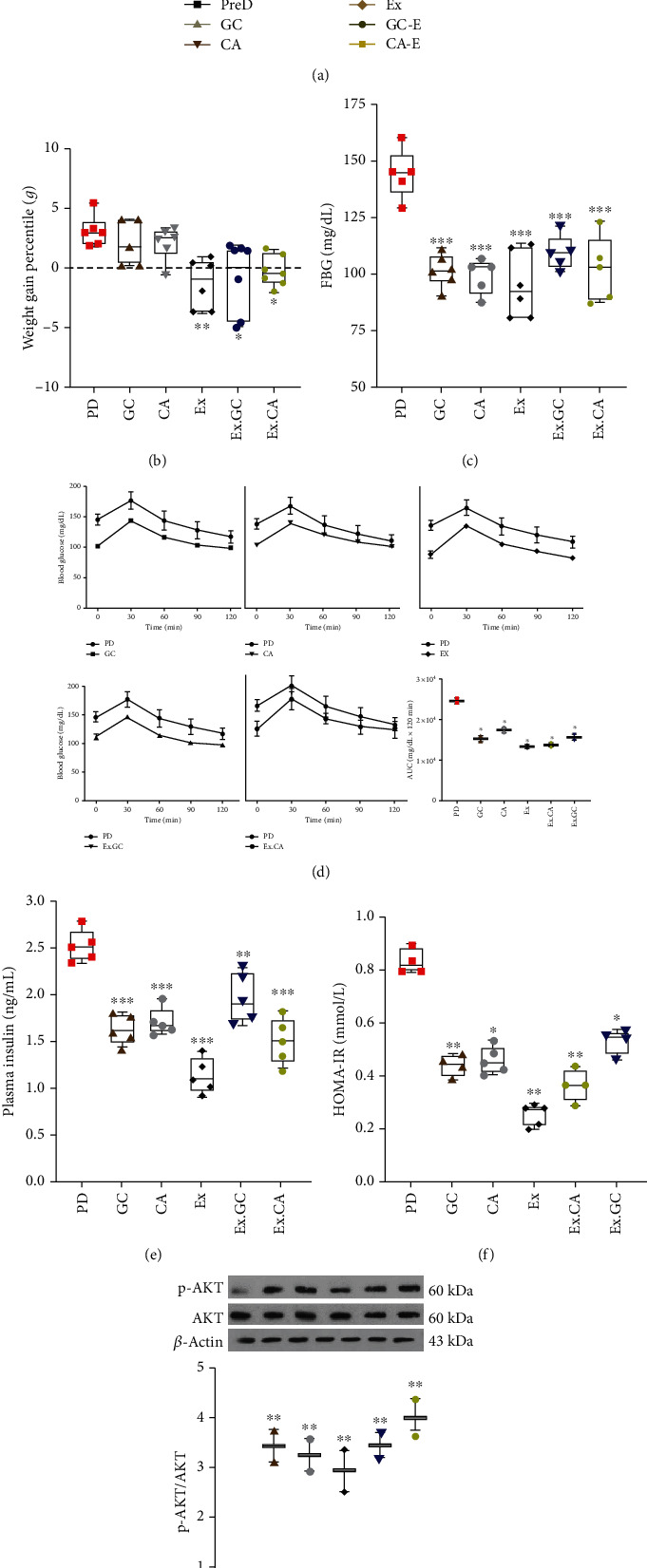
Several treatments mitigated HDF-associated metabolic changes. (a) Weight of mice in the PD, GC, CGA, EX, EX. GC, and EX.CGA groups. (b) The final weight of mice in the PD, GC, CGA, EX, EX. GC, and EX.CGA groups compared to weights at week 12. (c) Measurement of fasting blood glucose (FBG), (d) glucose tolerance test (GTT), and the area under the curve (AUC) in PD and all treated groups at the end of the 22^nd^ week. (e) Measurement of plasma insulin concentration and (f) HOMA index in PD and all treated groups at the end of the 22^nd^ week. (g) Immunoblot analysis of protein levels of p-AKT and total AKT in livers from PD and all treated group mice. All values are presented as mean ± SD. ^∗^ = *p* < 0.05, ^∗∗^ = *p* < 0.01, and ^∗∗∗^ = *p* < 0.001 indicate significant statistical differences in the treated compared to PD mice.

After induction of PD, the mice were treated with training (EX), green coffee (GC), chlorogenic acid (CGA), or a combination of these factors (EX+GC and EX+CGA). The GC used in this study contained 50% CGA and less than 2% caffeine, as revealed by HPLC (Figure 2). In the groups receiving GC and CGA, no weight loss was observed compared to the control PD group. In contrast, the mice that underwent EX regardless of other treatments showed significant weight loss compared to the control PD group (Figures 6(a) and 6(b)). All treatments improved blood glucose levels (Figure 6(c)) and glucose tolerance in the PD mice (Figure 6(d)). Moreover, all treatments resulted in decreased plasma insulin levels compared to the controls (Figure 6(e)). Importantly, EX was found to have the greatest effect on insulin levels. In keeping with these findings, the HOMA-IR index was lower in the treated PD mice compared to the untreated (Figure 6(f)).

**Figure 7:**
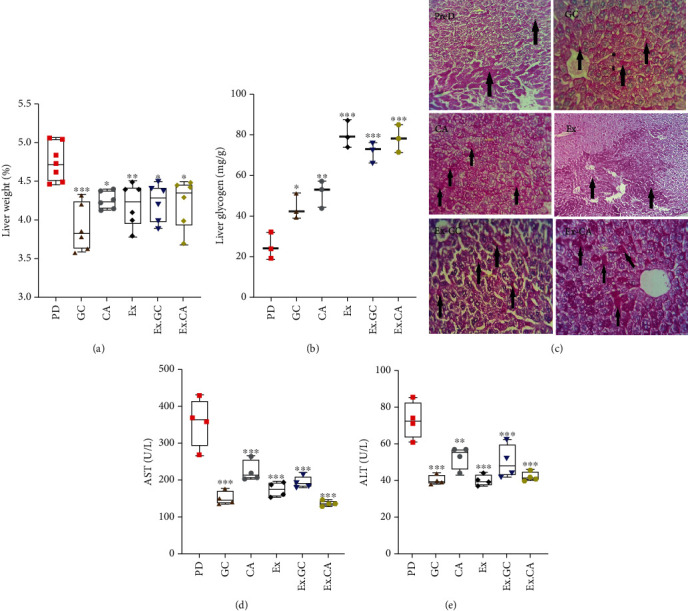
GC, CGA, EX, EX. GC, and EX.CGA treatment limited HFD-associated changes in murine livers. (a) The ratio of liver to total body weight in PD and all treated animal groups at the end of the 22^nd^ week. (b) Quantitative assessment of hepatic glycogen in the livers from the PD and all treated groups. (c) PAS staining of liver from PD and all treated animal groups (arrows indicate hepatic glycogen). (d and e) Plasma levels of AST and ALT in PD and all treated groups. All values are presented as mean ± SD. ^∗^ = *p* < 0.05, ^∗∗^ = *p* < 0.01, and ^∗∗∗^ = *p* < 0.001 indicate significant statistical differences in the treated compared to PD mice.

The accumulated protein level of hepatic AKT phosphorylation was higher in samples from all the intervention groups compared to the PD group (Figure 6(g)). This corresponded to the decreased liver to total body weight ratios in the treated compared to untreated PD mice. However, this trend was statically significant in the whole group-treated animals (Figure 7(a)). PAS staining in liver sections from treated PD mice was also judged less compared to the controls (Figures 7(b) and 7(c)). Similarly, serum levels of AST and ALT were decreased in all intervention groups (Figures 7(d) and 7(e)).

### 3.4. Training, Green Coffee, and Chlorogenic Acid Treatment Alter Hepatic mRNA Levels of Genes Associated with Glycogen Synthesis, Gluconeogenesis, and Glycolysis

**Figure 8:**
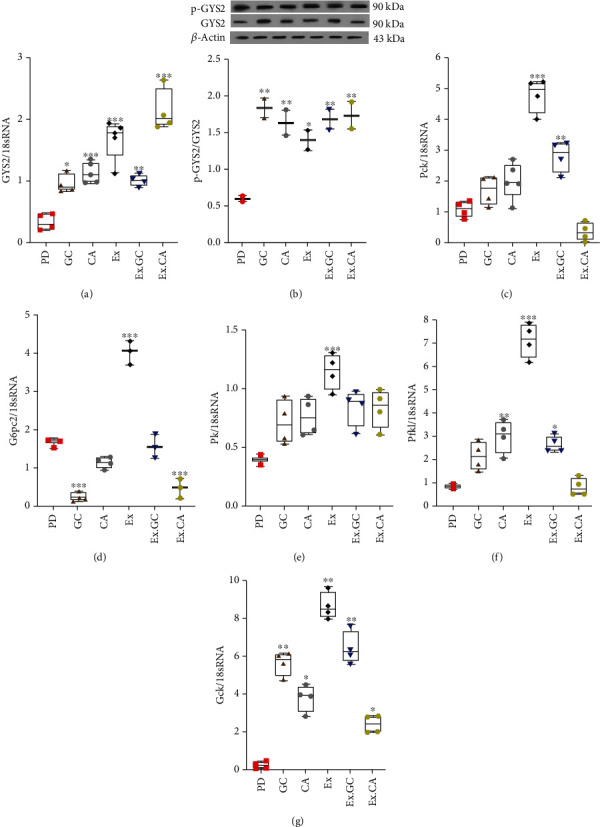
GC, CGA, EX, EX. GC, and EX.CGA treatment alters mRNA expression of multiple metabolic genes in livers from PD mice. (a) RT-qPCR analysis of *Gys2* mRNA levels in the liver from PD and all treated animal groups. (b) Immunoblot of protein levels of p-GYS2 and total GYS2 in the liver from PD and all treated groups. (c) RT-qPCR analysis of *G6pc2* and (d) *Peck* mRNA in the livers from PD and all treated animal groups. (e–g) RT-qPCR analysis for *Pk*, *Pfkl*, and *Gk* mRNA levels in the livers from PD and all treated groups. All values are presented as mean ± SD. ^∗^ = *p* < 0.05,^∗∗^ = *p* < 0.01, and ^∗∗∗^ = *p* < 0.001 indicate significant statistical differences in the treated compared to PD mice.

In all treatment groups, hepatic mRNA levels of *Gys2* and levels of phosphorylated Gys*2* were increased (Figures 8(a) and 8(b)) were decreased (Figure 8(b)) compared to the untreated PD group. mRNA levels of *Pck* were increased in the EX and EX+GC-treated PD mice versus the controls (Figure 8(c)). As well, EX was associated with increased hepatic *G6pc2* mRNA, whereas in animals treated with GC or EX+CGA groups mRNA levels were decreased (Figure 8(d)). Hepatic mRNA levels P*fkl*, *Gk*, and *Pk* were found to trend up in PD mice regardless of the intervention compared to the controls. However, the difference was only significant in the EX-treated animals (Figures 8(e) and 8(g)). mRNA levels of *Pfkl* were significantly increased in the CGA-, EX-, and EX+GC-treated groups (Figure 8(f)).

## 4. Discussion

In the current study, the effects of training in combination with green coffee and chlorogenic acid in the HFD-fed PD mice were evaluated. To the best of our knowledge, this is the first published study to address this. Consistent with the findings in people, the HFD-fed mice displayed metabolic dysregulation including elevated FBG, insulin and AST/ALT levels, abnormal glucose tolerance, and increased HOMA index. Additionally, increased liver weight, decreased glycogen levels, and AKT phosphorylation were observed. Furthermore, the HFD-fed mice showed disruption in hepatic glycogen synthesis as evinced by reduced expression of Gys2, increased gluconeogenesis via increased Pck and G6pc2, and increased glycolysis via decreased expression of Gk and Pk.

Therapeutic intervention with training, green coffee, or chlorogenic acid found that training was most efficacious at resisting HDF-mediated metabolic dysregulation including glucose balance, glycogen accumulation, and body and liver weight changes. This is consistent with clinical data that identified constant training as having an impact on metabolic syndrome and diabetes [27].

In mice, HFD is an accepted means of inducing metabolic imbalance (Xu, D., Jiang et al. in 2019, Asare-Bediako et al. in 2020 and Mu, H. N) [28–30]. EX, GC, and CGA independently reduce FBS and improved insulin resistance. We found that while EX caused weight loss in the PD mice, GC and CGA alone had no effect, as we found, others reported that EX promoted weight loss and improvement in body composition. However, there effects of GC and CGA on weight loss are controversial [17, 20, 31–33]. It is possible that variation in study design and dosages explain variation in reported outcomes.

The liver controls blood glucose levels via the regulation of anabolic (glycogen synthesis and gluconeogenesis) and catabolic (glycolysis and glycogenolysis) pathways. By activation of PI3K-AKT signalling pathway, insulin causes progression of glycogen synthesis and gluconeogenesis and inhibits glycolysis and glycogenolysis. In PD and T2D, liver glycogen storage is dysregulated. Conversion of glucose to glycogen is performed by GYS2. In a feedback manner, GYS2 is activated by the deactivation of GSK-3 through the PI3K-AKT pathway. Based on our results, the HFD feeding reduced hepatic AKT phosphorylation and decreased *Gys2* expression. In contrast, several treatments were associated with increased hepatic PI3K-AKT-GYS2 expression. These data were confirmed [34, 35]. We believe that our interventions were effective in glycogen synthesis, which can cause glycogen to store blood glucose in the liver (Figures 8(a) and 8(b)). Inhibition of liver glucose production by insulin occurs, in part, via PI3K-AKT-mediated suppression of PCK1and G6PC2. We found that in the PD group, subsequent to changes in insulin levels, the expressions of the gluconeogenesis-regulating factors, Pck and G6pc2, were decreased. The interactions between training and gluconeogenesis are also controversial. Some data suggest increased hepatic gluconeogenesis, while others suggest decreased hepatic gluconeogenesis after training. We noted increased *Pck* expression after training verifying other reports. [36, 37]. In the path of gluconeogenesis in training interventions genes involved such as G6pc2, Pck increase due to the body physiological conditions and the body's need for energy during the endurance activity with high intensity. But this increase has been enough to meet the body's need for energy not to increase glucose in the blood. Decreased activity of PK and PFK, which occurs in diabetes, may decrease glycolysis and glucose consumption. Herein, we found all three enzymes were decreased in the livers from the PD mice compared to the controls, in keeping with other reports [38–40]. Additionally, all interventions led to increased expression of *Gk* and *Pk*, although changes *Pfk1* expression were found only in PD mice treated with the EX, EX+GC, and CGA groups. However, there may be other potentially unknown mechanisms by which these interventions improve metabolic status in prediabetic individuals, which requires further research.

The current study has a number of limitations. First, the animal cohorts were young and PD and metabolic syndrome and diabetes are largely found in mid and older age adults. Second, the study tested interventions on of PD-related changes but was not blinded in its design. While training would not be blinded, the other agents could have been administered in a blinded manner. Third, the data is associative and does not demonstrate a necessary link between intervention and outcome. Fourth, the study design did not consider the effect of sex upon the outcomes. Additionally, changes in tissue mRNA levels from whole organs are neither cell specific nor do they show cell signalling or metabolite variation. Finally, the overall length of the study interval was brief. PD, metabolic syndrome, and diabetes are chronic conditions.

In summary, the results of this study demonstrated that aerobic training in conjunction with green coffee lowered blood glucose and mitigated metabolic changes associated with PD in mice. These data support further study looking at long-term effects with a goal of possible translational applications.

## Figures and Tables

**Table 1:** PCR primers.

Gene	Forward primer (5′-3′)	Reverse primer (5′-3′)	Annealing temperature (°C)
GYS2	ACCAAGGCCAAAACGACAG	GGGCTCACATTGTTCTACTTGA	58
PCK	AAGCATTCAACGCCAGGTTC	GGGCGAGTCTGTCAGTTCAAT	56
G6PC2	CCTGATGGTGGTGGCTCTA	TCTCTGTGCTGTGGCTCTATT	56
PK	ATATCACCCAGGTCGTTGCA	CGAAGCGCAGATCCAAAAGA	54
PFKL	GTGGAAGGAGGCGAGAACATCAAG	GCCGTGTTGGAGCAGATTGTAGG	60
GC	CCCTGTAAGGCACGAAGACATA	AGAAGTCCCACGATGTTGTT	56

## Data Availability

Data are available on request.

## Abbreviations

ADA: American Diabetes Association
AKT: Protein kinase B
ALT: Alanine aminotransferase
AST: Aspartate aminotransferase
BSK: Bonyan Salamat Kasra
CHA: Chlorogenic acid
EX: Training training
FBG: Fasting blood glucose
FOXO1: Forkhead box protein O1
G6PC2: Glucose-6-phosphatase 2
GC: Green coffee
GK: Glucokinase
GSK3: Glycogen synthase kinase 3
GTT: Glucose tolerance test
GYS2: Glycogen synthase 2
H&E: Hematoxylin and eosin
HFD: High-fat diet
HOMA: Homeostatic model assessment of insulin resistance
IFG: Impaired fasting glucose
IGT: Impaired glucose tolerance
IRS1/2: Insulin receptor substrate 1
MAV: Micro aerial vehicle
PAS: Periodic acid-Schiff
PD: Prediabetes
PEPCK: Phosphoenolpyruvate carboxykinase
PFKL: Phosphofructokinase, liver type
PI3K: Phosphoinositide-3 kinase
PK: Pyruvatekinase
T2DM: Diabetes mellitus type 2.

## Additional Points

*New & Noteworthy.* (i) Training, green coffee, chlorogenic acid, and the combination of training with green coffee and chlorogenic acid can improve the symptoms and complications of prediabetes by regulating the glycogen synthesis mechanism by activating the signalling pathway of IRS1, PI3K, AKT, GSK3, and GYS2. The results were somewhat effective. The results were so effective that blood sugar, plasma insulin, and liver enzymes were in the normal range. (ii) Training, green coffee, and chlorogenic acid can improve prediabetic conditions by activating genes involved in glycolysis mechanisms, such as PK, PFKL, and GK, and prevent the disease from progressing to type 2 diabetes. (iii) Green coffee and a combination of training and chlorogenic acid, by controlling the genes involved in the mechanism of gluconeogenesis, interfere with the activity of this signalling pathway and thus can prevent the production of glucose and glucose accumulation in the blood.
